# Natural convection of $\mathrm {Al}_{2}\mathrm {O}_{3}$-water nanofluid in a non-Darcian wavy porous cavity under the local thermal non-equilibrium condition

**DOI:** 10.1038/s41598-020-75095-5

**Published:** 2020-10-22

**Authors:** Ammar I. Alsabery, Tahar Tayebi, Ali J. Chamkha, Ishak Hashim

**Affiliations:** 1 Refrigeration and Air-conditioning Technical Engineering Department, College of Technical Engineering, The Islamic University, Najaf, Iraq; 2 Department of Mathematical Sciences, Faculty of Science and Technology, Universiti Kebangsaan Malaysia (UKM ), 43600 Bangi Selangor, Malaysia; 3 Faculty of Sciences and Technology, Mohamed El Bachir El Ibrahimi University, Bordj Bou Arreridj, El-Anasser, Algeria; 4 Energy Physics Laboratory, Department of Physics, Faculty of Science, Mentouri Brothers Constantine 1 University, Constantine, Algeria; 5 Department of Mechanical Engineering, Prince Sultan Endowment for Energy and the Environment, Prince Mohammad Bin Fahd University, Al-Khobar, 31952 Saudi Arabia; 6 RAK Research and Innovation Center, American University of Ras Al Khaimah, P.O. Box 10021, Ras Al Khaimah, United Arab Emirates

**Keywords:** Mechanical engineering, Applied mathematics

## Abstract

This study investigates thermal natural convective heat transfer in a nanofluid filled-non-Darcian porous and wavy-walled domain under the local thermal non-equilibrium condition. The considered cavity has corrugated and cold vertical walls and insulated horizontal walls except the heated part positioned at the bottom wall. The transport equations in their non-dimensional model are numerically solved based on the Galerkin finite-element discretization technique. The dimensionless governing parameters of the present work are the nanoparticle in volume concentration, the Darcy number, number of undulations, modified heat conductivity ratio, dimensionless heated part length, and location. Comparisons with other published theoretical and experimental results show good agreement with the present outcomes. The findings indicate that the heater length, its position, and the waves number on the side vertical walls as well as the nanoparticles concentration can be the control parameters for free convective motion and heat transport within the wavy cavity.

## Introduction

Natural convection heat exchange mode in cavities with various configurations represents a vital role in maximizing energy production and reducing energy losses in many engineering applications, such as cooling the minuscule devices of electronic systems, air conditioning systems, lubrication systems, exploitation of solar energy, heat storage containers, and so on. In most of these frameworks, traditional liquids such as water and oils have been used as a heat transfer agent. However, the limitations of the heat transfer enhancement using such a traditional liquid have been reached due to the low thermal conductivity. Researchers have found a new path by suspending nano-sized metallic or metal oxides solid materials in the host-liquids where an increment of thermal conductivity was conducted. This idea gave rise to the nascence of the so-called “Nanofluid”. Nanofluids are usual liquids that contain usually nano-scaled (less than 100nm) metal or metal oxide suspended materials like Cu, Ag, CuO, $\mathrm {Al}_{2}\mathrm {O}_{3}$, $\hbox {TiO}_2$, $\hbox {SiO}_2$ and ZnO.

Nanofluids have a more important overall heat conductivity than the usual liquids. Applications of nanofluids can be found in electronic and medical industries. In this regard, many research works have been published in the literature as an attempt to understand heat transfer enhancement in nanofluids-filled cavities. The buoyancy-driven heat exchange enhancement in a nanofluids-filled enclosure is investigated firstly by Khanafer et al.^1^. Jou and Tzeng^2^ adopted Khanafer’s nanofluid model^1^ to investigate the nature of the convective heat exchange increase of a nanofluid-filled 2D enclosure. A boost in the average heat transfer coefficient was achieved by increasing the buoyancy factor and concentration in volume of nanoparticles. Öztop and Abu-Nada^3^ also investigated the thermal free convection in a partially heated rectangular cavity filled with nanofluids. It was found that the convection heat transfer was improved with the insertion of nanoparticles. Mahmoudi et al.^4^ have simulated natural convection heat transfers in a nanofluid-filled square domain with a heat source that was attached horizontally. The effects of a magnetic field on thermal free convection in CuO-water nanoliquid-filled sinusoidally-walled cavity were perused by Sheikholeslami et al.^5^. Hu et al.^6^ concluded that the thermal free convection of $\hbox {TiO}_2$-$\hbox {H}_2$O based nanoliquid was enhanced with the addition of the nano-sized solid particles. Alsabery et al.^7^ studied transient natural-convective flow and heat exchange characteristics under the effects of non-uniform thermal boundary conditions of a nanofluid-filled trapezoidal chamber. They reported that the best heat exchange enhancement was obtained by the larger side wall inclination angle and a higher concentration in volume of the used solid nanomaterials. For good reviews on the fundamental theory and applications of nanofluid flows and heat transfer the readers can refer the works of Mahian et al.^8,9^.

Most often, the overall nanoliquid heat conductivity and viscosity of are evaluated utilizing the Maxwell-Garnett and Brinkman theoretical models, respectively. A new model inspired by experimental data has been proposed by Corcione^10^ to evaluate these properties. Motlagh and Soltanipour^11^ investigated arithmetically using the Corcione’s nanofluid model^10^ the thermal natural convection characteristics in a nanofluid-filled square domain using the two-phase approach. The results revealed that the nanofluid enhances the heat exchange rate and positively depends on the increment of solid particle volume fraction. Tayebi and Chamkha^12,13^ described the thermal-free-convective motion, entropy production behaviour and heat exchange rate of a hybrid nanoliquid-filled square domain with different inserted conductive bodies employing Corcione’s nanofluid model. Using the same nanofluid model^10^, Alsabery et al.^14^ examined through a numerical code the conjugate heat transfer in a square cavity having a concentric solid insert. The $\mathrm {Al}_{2}\mathrm {O}_{3}$-water nanoliquid with different values of volume fraction is used.

Thermal convection within porous mediums has many practical applications, such as petroleum processing, groundwater flows, filtering, food processing, and fibrous insulation^15^. There are two different models for the heat equation related to the two phases of the porous matrix. The first one is the so-called local thermal equilibrium (LTE) model which assumes that both the solid and fluid temperatures are the same. The literature on this model is abundant. Basak et al.^16^ have perused the effects of several types of thermal boundary conditions on thermal free convection in a porous domain. Zahmatkesh^17^ also investigated the significance of thermal boundary conditions in heat transfer to cause natural convection and entropy generation in a square porous domain heated from the bottom. He found that the heat exchange boosted towards the center of the cavity. Simultaneous thermal free convection and entropy production behavior in porous rhombic cavities were investigated by Anandalakshmi and Basak^18^. Sheikholeslami^19^ considered the problem of CuO-water nanoliquid natural convection in a porous cavity using the Darcy law model. In the research work of Rashad et al.^20^ , the impacts of the sink/source position and enlargement on the entropy production and free convective motion under a magnetic excitation in a titled porous domain saturated with a Copper-water nanoliquid are interpreted. Since the LTE approach is not feasible under some conditions in which the solid and fluid temperatures are different, a second model so-called the local thermal non-equilibrium (LTNE) was introduced. The LTNE model is applicable in many applications such as food drying/freezing, microwave heating, and cooling of microelectronics devices^21^.

Theoretical and experimental studies adopting the LTNE approach in porous mediums are still limited. The problem of thermal-natural convection in a porous enclosure taking into consideration the LTNE approach was studied numerically by Baytaş and Pop^22^. Baytaş^23^ used the LTNE model to investigate natural convection in a non-Darcian square porous cavity having a heat-generating solid phase. They concluded that when the heat transfer coefficient is small, then the temperature of the fluid phase was lower than that of the solid phase. Badruddin et al.^24^ also conducted a study on the effect of thermal-natural convection in a square porous cavity using LTNE approach. The onset of convection in a nanofluid saturated porous layer employing the LTNE approach was studied by Kuznetsov and Nield^25^ and Bhadauria and Agarwal^26^. Sheremet et al.^27^ examined numerically the effect of the LTNE approach on thermal-natural convection in a nanoliquid saturated-porous enclosure. The study concluded that lower inter-phase heat transfer coefficient can decrease the heat exchange rate. Considering the LTNE condition and Buongiorno’s model, Zargartalebi et al.^28^ have investigated numerically the unsteady-state thermal free convection in a nanoliquid-saturated porous enclosure. It was found that the average heat exchange can be enhanced by reducing the buoyancy ratio and increasing the Rayleigh number. Baytaş and Baytaş^29^ perused the effect of LTNE model on thermal free convection in a square domain in the presence of internal heat generation of a porous layer. Alsabery et al.^30^ investigated the non-uniform heating and wall conduction effects on thermal free convection in a square porous cavity based on the LTNE approach. Tahmasebi et al.^31^ explained the effect of LTNE model on the natural convective heat transfer in a cavity that partially filled with porous media with the use of Buongiorno’s nanofluid model. They observed that with the increasing of the interface heat transfer parameter, the overall exchange rate of the nanoliquid phase was reduced. Sheremet and Pop^32^ applied the finite difference method to investigate the effects of the size and position of a local heater on the natural convection in a square porous cavity based on the LTNE and Buongiorno’s models. Izadi et al.^33^ perused the effect of the LTNE condition on convective heat transfer in a square porous cavity filled with CuO-water micropolar nanofluids.

The complex geometry of cavities is an important aspect that can reflect its existence in many engineering applications such as heat exchangers, devices of food treatment, refineries, refrigeration systems, condensates and so on. Therefore, several researches have been conducted involving thermal convection problems in complex cavities. Kumar^34^ computationally explained the thermal free convection and fluid motions within a wavy cavity that filled with a porous medium. The results indicated that convective exchanges were clearly affected by Rayleigh number, undulations ‘amplitude, phase of wave, and undulations number of the vertical-cavity walls. Das and Mahmud^35^ have examined the thermal natural convection within a double wavy-walled domain. Their results suggested that heat exchange was highly influenced by the amplitude of the wavy wall and the number of undulations. Misirlioglu et al.^36^ analyzed the effect of internal heat generation on thermal free convection in a porous square cavity using the Darcy model. Thermal free convection in a porous-undulated enclosure based on the Darcy–Brinkman–Forchheimer extended model is computationally studied by Chen et al.^37^. It was found that local heat exchange dependent on the Darcy number and the porosity of the media. Fluid flow due to thermal free convection in a non-Darcian wavy-walled porous enclosure was considered by Khanafer et al.^38^. The results depicted that the convective flow circulation within the examined enclosure was boosted with rising Rayleigh number. Using the finite volume method, Abu-Nada and Öztop^39^ investigated the thermal convection within a wavy-walled enclosure occupied with $\mathrm {Al}_{2}\mathrm {O}_{3}$-$\hbox {H}_2$O nanoliquids. They noticed that the insertion of solid nanoparticles into the water (based fluid) makes the convective heat exchange in the domain enhances. Combined free and forced thermal convection in a nanofluid-filled lid-driven wavy-walled enclosure was carried out by Abu-Nada and Chamkha^40^. They explained that the convective exchange was positively increases with increasing nanoparticles concentration (in volume) for all Richardson numbers. Sheremetet al.^41^ have worked on an open nanolliquid-filled wavy porous enclosure to investigate the unsteady-state free convection using Buongiorno’s model. Using the same model of nanofluid, Sheremet and Pop^42^ investigated the influence of non-uniform temperature conditions on thermal natural convection in a wavy-walled porous enclosure. Additionally, Alsabery et al.^43^ examined numerically the entropy production and convective heat exchange in a wavy-walled porous enclosure having a rotating rigid cylinder and heated from below. They concluded that an increment in the porosity of the medium caused an enhancement in the convection heat transfer. Alsabery et al.^44^ studied the impact of the two-phase nanofluid technique and the role of the heat source or sink on thermal natural convection in a square cavity with a rigid cylinder.

According to the studies discussed above and to our best knowledge, it can be noticed that there is no research about the problem of thermal free convection in a wavy-porous cavity that filled with regular fluids or nanofluids considering the local thermal non-equilibrium condition. Thus, this is an original piece of work and potentially a valuable source. Consequently, the aim of this study is to evaluate the flow motions and thermal natural convective heat exchanges of $\mathrm {Al}_{2}\mathrm {O}_{3}$-$\hbox {H}_2$O-based nanoliquid in a non-Darcian wavy-porous cavity under the local thermal non-equilibrium condition. The findings of such a work may have possible applications in many practical systems such as heat exchangers, solar-energy devices, food treatment systems, refineries, refrigeration systems, condensates, and so on.

## Mathematical formulation

**Figure 1:**
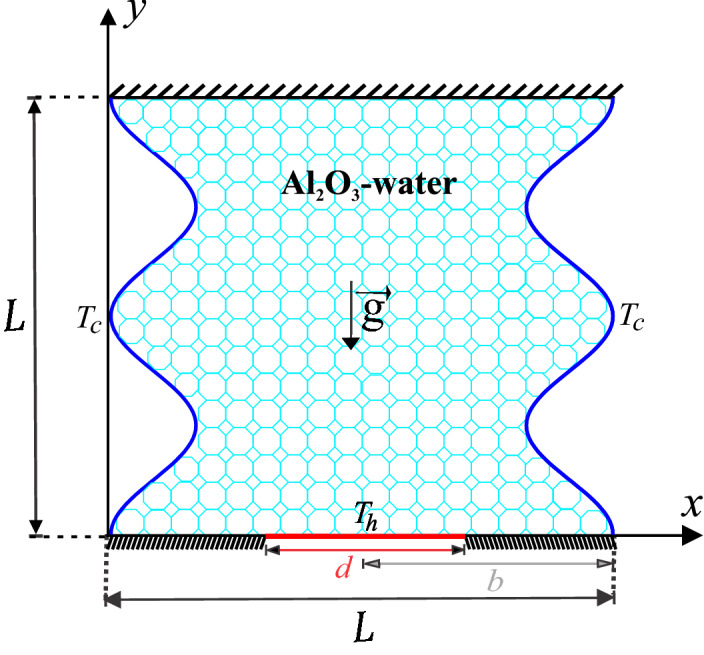
Problem geometry.

Figure 1 shows the problem geometry that will be considered in this work. An isothermal heater is put in place within the horizontal bottom wall with length *d*, whereas the vertical wavy walls are cold ($T_c$). The rest of the walls are adiabatic. The porous cavity is filled with $\mathrm {Al}_{2}\mathrm {O}_{3}$-water nanofluid. The Forchheimer-Brinkman-extended Darcy model are adopted together with the Boussinesq approximation. The type of the porous media used is glass balls. Considering the local thermal non-equilibrium (LTNE) model, the governing equations are as follows

1 $$\begin{aligned}&\frac{{\partial u}}{{\partial x}} + \frac{{\partial v}}{{\partial y}} = 0, \end{aligned}$$

2 $$\begin{aligned}&\frac{\rho _{nf}}{\varepsilon ^2} \left( u\frac{{\partial u}}{{\partial x}} + v\frac{{\partial u}}{{\partial y}}\right) = - \frac{{\partial p}}{{\partial x}} + \frac{\mu _{nf}}{\varepsilon }\,\left( {\frac{{{\partial ^2}u}}{{\partial {x^2}}} + \frac{{{\partial ^2}u}}{{\partial {y^2}}}} \right) -\left( \frac{\mu _{nf}}{K}u-\frac{1.75}{\sqrt{150}\varepsilon ^{3/2}} \frac{\rho _{nf} u \left| \mathbf{u} \right| }{\sqrt{K}} \right) , \end{aligned}$$

3 $$\begin{aligned}&\frac{\rho _{nf}}{\varepsilon ^2} \left( u\frac{{\partial v}}{{\partial x}} + v\frac{{\partial v}}{{\partial y}}\right) = - \frac{{\partial p}}{{\partial y}} + \frac{\mu _{nf}}{\varepsilon }\,\left( {\frac{{{\partial ^2}v}}{{\partial {x^2}}} + \frac{{{\partial ^2}v}}{{\partial {y^2}}}} \right) -\left( \frac{\mu _{nf}}{K}v-\frac{1.75}{\sqrt{150}\varepsilon ^{3/2}} \frac{\rho _{nf} v \left| \mathbf{u} \right| }{\sqrt{K}} \right) + (\rho \beta )_{nf} \text {g}(T_{h} - {T_c}), \end{aligned}$$

4 $$\begin{aligned}&u\frac{{\partial T_{nf}}}{{\partial x}} + v\frac{{\partial T_{nf}}}{{\partial y}} = \frac{\varepsilon k_{nf}}{(\rho C_{p})_{nf}} \left( {\frac{{{\partial ^2}T_{nf}}}{{\partial {x^2}}} + \frac{{{\partial ^2}T_{nf}}}{{\partial {y^2}}}} \right) + \frac{h\left( {T_{s} - T_{nf}} \right) }{(\rho C_{p})_{nf}}, \end{aligned}$$

5 $$\begin{aligned}&0 = (1 - \varepsilon ) k_{s} \left( {\frac{\partial ^2 T_{s}}{{\partial {x^2}}} + \frac{\partial ^2 T_{s}}{\partial y^2}} \right) + h\left( {T_{nf} - T_{s}} \right) , \end{aligned}$$

where $\left| \mathbf{u} \right| =\sqrt{u^2 + v^2}$ represents the Darcy velocity, $\text {g}$ is the gravitational acceleration, $\varepsilon$ the medium’s porosity, $K = \frac{\varepsilon ^{3} d^2_m}{150 (1-\varepsilon )^2}$ is the medium’s permeability, with $d_m$ representing the porous bed’s average particle size.

The following nanofluid thermophysical properties are utilized:

6 $$\begin{aligned}&\rho _{nf}=(1-\phi )\rho _{f}+\phi \rho _{p}, \quad {(\rho \beta )_{nf}} = (1 - \phi ){(\rho \beta )_{f}} + \phi {(\rho \beta )_{p}}, \end{aligned}$$

7 $$\begin{aligned}&(\rho C_p)_{nf}=(1-\phi )(\rho C_p)_{f}+\phi (\rho C_p)_{p}, \quad \alpha _{nf}=\frac{k_{nf}}{(\rho C_p)_{nf}}, \end{aligned}$$

where $\phi$ is the nanoparticle volume fractions. The nanofluid’s ratios of thermal conductivity and dynamic viscosity are^10^

8 $$\begin{aligned} \frac{k_{nf}}{k_{f}}= 1 + 4.4 \text {Re}_B^{0.4} \text {Pr}^{0.66} \left( \frac{T}{T_{fr}}\right) ^{10} \left( \frac{k_p}{k_f}\right) ^{0.03} \varphi ^{0.66}, \quad \frac{\mu _{nf}}{\mu _f} = (1-34.87(d_p/d_f)^{-0.3}\varphi ^{1.03})^{-1}, \end{aligned}$$

where $\text {Re}_B=\rho _f u_B d_p/\mu _f, \, u_B = 2k_b T/(\pi \mu _f d_p^2).$ Further details can be found in Corcione^10^.

Next the following dimensionless variables are utilized:

9 $$\begin{aligned} X,Y= & {} \frac{x,y}{L},\,\, U,V=\frac{uL,vL}{\alpha _f},\,\, \theta _{nf}=\frac{T_{nf} - T_c}{T_h - T_c},\,\, \theta _s=\frac{T_s - T_c}{T_h - T_c},\,\, \Pr = \frac{\nu _f}{\alpha _f},\nonumber \\ Ra= & {} \frac{g \beta _f \left( T_h-T_c \right) L^3}{\nu _{f} \alpha _{f}}, \,\, P=\frac{pL^2}{\rho _{f} \alpha _f^2}, \,\, k_{eff}=\varepsilon k_{nf}+(1-\varepsilon )k_m, \,\, C_F = \frac{1.75}{\sqrt{150}}. \end{aligned}$$

As a results we have

10 $$\begin{aligned}&\frac{{\partial U}}{{\partial X}} + \frac{{\partial V}}{{\partial Y}} = 0, \end{aligned}$$

11 $$\begin{aligned}&\frac{1}{\varepsilon ^2}\left( U\frac{\partial U}{\partial X} + V\frac{\partial U}{\partial Y}\right) = - \frac{\partial P}{\partial X}+ \frac{\rho _{f}}{\rho _{nf}} \frac{\mu _{nf}}{\mu _{f}} \frac{\Pr }{\varepsilon }\left( \frac{\partial ^2 U}{\partial X^2} + \frac{\partial ^2 U}{\partial Y^2}\right) - \frac{\rho _{f}}{\rho _{nf}} \frac{\mu _{nf}}{\mu _{f}} \frac{\Pr }{Da}U- \frac{C_F\sqrt{U^2 + V^2}}{\sqrt{Da}} \frac{U}{\varepsilon ^{3/2}}, \end{aligned}$$

12 $$\begin{aligned}\frac{1}{\varepsilon ^2}\left( U\frac{\partial V}{\partial X} + V\frac{\partial V}{\partial Y}\right) &= - \frac{\partial P}{\partial Y}+ \frac{\rho _{f}}{\rho _{nf}} \frac{\mu _{nf}}{\mu _{f}} \frac{\Pr }{\varepsilon }\left( \frac{\partial ^2 V}{\partial X^2} + \frac{\partial ^2 V}{\partial Y^2}\right) - \frac{\rho _{f}}{\rho _{nf}} \frac{\mu _{nf}}{\mu _{f}} \frac{\Pr }{Da}V- \frac{C_F\sqrt{U^2 + V^2}}{\sqrt{Da}} \frac{V}{\varepsilon ^{3/2}} \nonumber \\&\quad + \frac{(\rho \beta )_{nf}}{\rho _{nf} \beta _{f}} {Ra}\, {\Pr }\, {\theta }, \end{aligned}$$

13 $$\begin{aligned}&\frac{1}{\varepsilon }\left( U\frac{{\partial \theta _{nf}}}{{\partial X}} + V\frac{{\partial \theta _{nf}}}{{\partial Y}}\right) = \frac{k_{eff}}{k_{f}} \frac{(\rho C_p)_{f}}{(\rho C_p)_{nf}} \left( \frac{{{\partial ^2}\theta _{nf}}}{{\partial {X^2}}} + \frac{{{\partial ^2}\theta _{nf}}}{{\partial {Y^2}}}\right) + \frac{(\rho C_p)_{f}}{(\rho C_p)_{nf}} H\left( {\theta _{s} - \theta _{nf}} \right) , \end{aligned}$$

14 $$\begin{aligned}&0=\frac{{{\partial ^2}\theta _s}}{{\partial {X^2}}} + \frac{{{\partial ^2}\theta _s}}{{\partial {Y^2}}}+ \gamma H \left( {\theta _{nf} - \theta _{s}} \right) , \end{aligned}$$

where the modified conductivity ratio $\gamma =\frac{\varepsilon k_{f}}{(1-\varepsilon ) k_{s}}$ and the inter-phase heat transfer coefficient $H=\frac{hL^{2}}{\varepsilon k_{f}}$. The boundary conditions now become

15 $$\begin{aligned}&U = V = 0,\,\, \frac{\partial \theta _{nf}}{\partial Y}=\frac{\partial \theta _{s}}{\partial Y}=0,\,\, 0\le X \le 1,\,\, Y=1, \quad \text {(top wall)}\end{aligned}$$

16 $$\begin{aligned}&U = V = 0,\,\, \theta _{nf}=\theta _{s}=1, \,\, Y=0, \,\, B-(0.5D)\le X \le B+(0.5D), \quad \text {(heated part of bottom wall)} \end{aligned}$$

17 $$\begin{aligned}&U = V = 0,\,\, \frac{\partial \theta _{nf}}{\partial Y}=\frac{\partial \theta _{s}}{\partial Y}=0,\,\, Y=0, \,\, 0\le X \le B-(0.5D), \,\, B+(0.5D)\le X \le 1, \quad \text {(rest of bottom wall)} \end{aligned}$$

18 $$\begin{aligned}{\,} & U = V = 0,\,\, \theta _{nf}=\theta _{s}=0,\,\, 1-A(1-\cos (2N\pi X)),\,\, 0\le Y\le 1, \quad \text {(left wall)} \end{aligned}$$

19 $$\begin{aligned}{} & U = V = 0,\,\, \theta _{nf}=\theta _{s}=0,\,\, A(1-\cos (2N\pi X)),\,\, 0\le Y\le 1, \quad \text {(right wall).} \end{aligned}$$

The local Nusselt and average numbers of the solid and nanofluid phases at the heated portion of the bottom horizontal wall are defined as

20 $$\begin{aligned} Nu_{s}= & {} \frac{k_{s}}{k_{f}} \left( \frac{\partial \theta _{s}}{\partial Y}\right) _{Y=0}, \quad Nu_{nf} = \frac{k_{eff}}{k_{f}} \left( \frac{\partial \theta _{nf}}{\partial Y}\right) _{Y=0}, \quad \nonumber \\ \overline{Nu}_{s}= & {} \int _{B-(0.5D)}^{B+(0.5D)} Nu_{s} {} \mathrm{{d}}X, \quad \overline{Nu}_{nf} = \int _{B-(0.5D)}^{B+(0.5D)} Nu_{nf} {} \mathrm{{d}}X. \end{aligned}$$

## Method of solution

Equations (10)–(14) subject to Eqs. (15)–(19) will be solved using the Galerkin weighted residual FEM. Hashim et al.^45^ gave a brief description of the method as follows: For each of the flow variables falling under the computational domain, different orders of Triangular Lagrange finite elements are employed. For every conservation equation, obtaining of residuals is done by replacing the approximations into the governing equations. A Newton–Raphson iteration algorithm is employed to simplify the nonlinear terms for the momentum equations. It is assumed that the convergence of the solution exists when the following convergence criteria are fulfilled by the relative error associated with each of the variables: $\left| (\Gamma ^{i+1}-\Gamma ^{i})/\Gamma ^{i+1}\right| \le \eta ,$ where *i* is the iteration number and $\eta$ is set at $\eta =10^{-6}$ in the present work. Based on the grid sensitivity checks presented in Table 1, for all computations in this paper, the G6 uniform grid is employed.

**Table 1:** Grid sensitivity checks: $\Psi_{\mathrm{min}}$, $\overline{Nu}_{nf}$ and $\overline{Nu}_{s}$ for $Ra=10^6$, $H=10$, $B=0.5$, $Da=10^{-3}$, $\phi =0.02$, $N=2$, $\gamma =10$, $D=0.5$.

Size of grid	# of elements	$\Psi_{\mathrm{min}}$	$\overline{Nu}_{nf}$	$\overline{Nu}_{s}$
G1	2971	$-2.5253$	3.9993	2.9993
G2	3403	$-2.5422$	4.0531	3.0531
G3	3909	$-2.5707$	4.0668	3.0668
G4	4810	$-2.59014$	4.1051	3.1051
G5	11794	$-2.6276$	4.2542	3.2542
G6	27151	$-2.6407$	4.2552	3.2552
G7	32745	$-2.6443$	4.2562	3.2562

**Figure 2:**
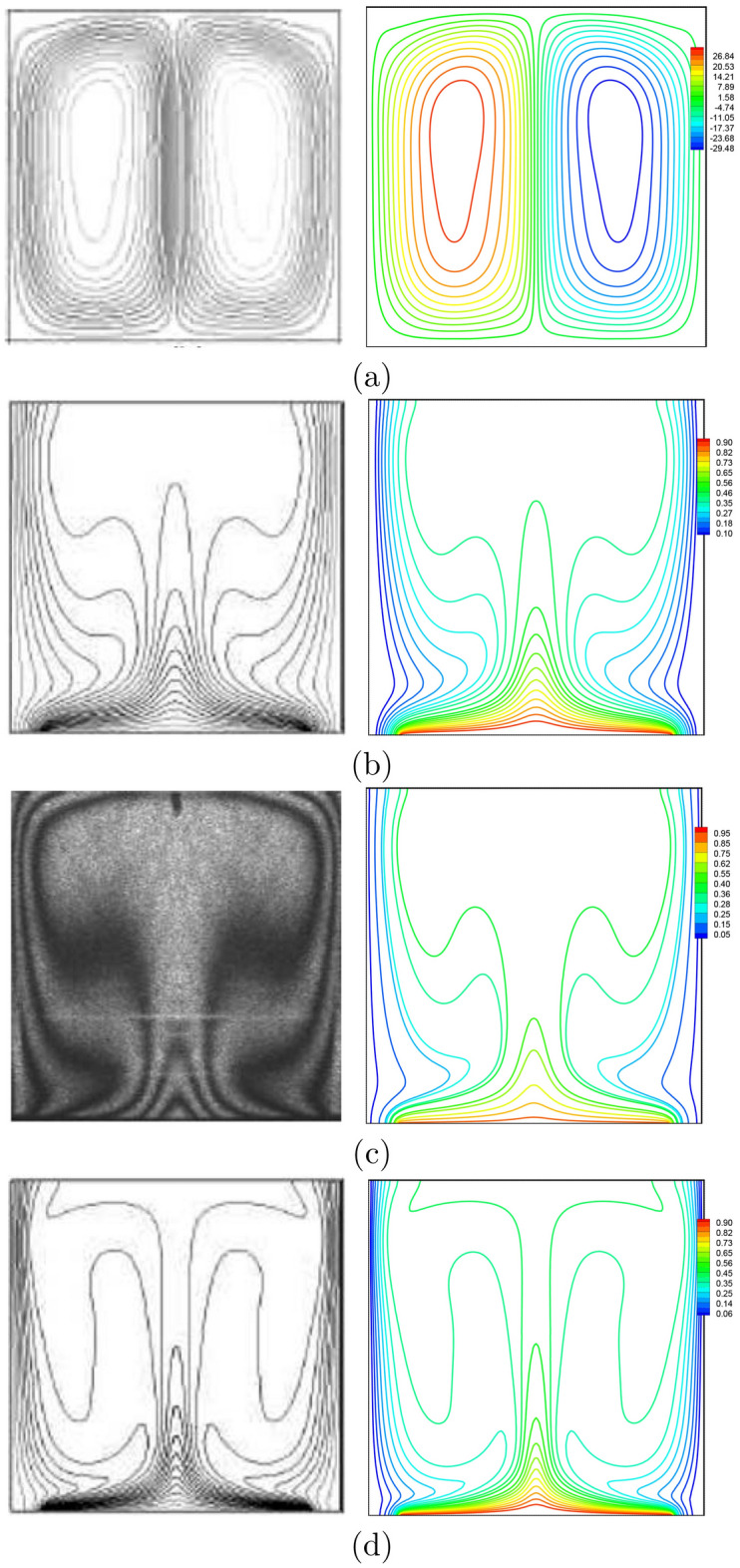
Comparison of (left) numerical and experimental results of Calcagni et al.^46^ and (right) present study: **(a)** streamlines ($Ra=10^{6}$, $D=0.4$), **(b)** isotherms ($Ra=10^{5}$, $D=0.8$), **(c)** isotherms ($Ra=1.836\times 10^{5}$, $D=0.8$), **(d)** isotherms ($Ra=10^{6}$, $D=0.8$) for $\phi =0$, $N=0$, $D=0$.

**Figure 3:**
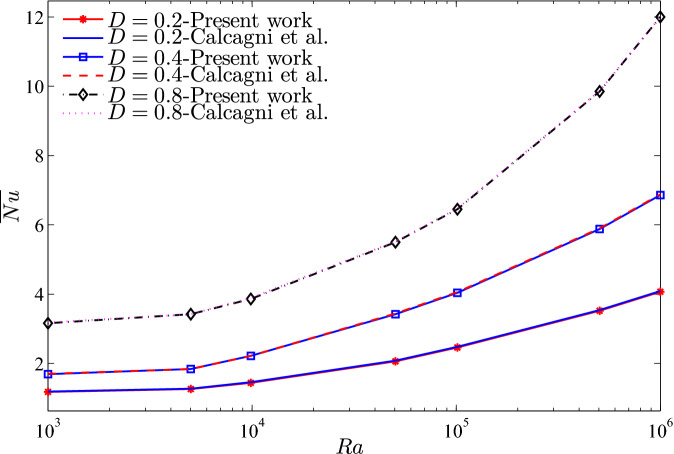
$\overline{Nu}$ vs. *Ra*: present study and Calcagni et al.^46^ for several *D* ($\phi =0$, $N=0$, $D=0$).

**Figure 4:**
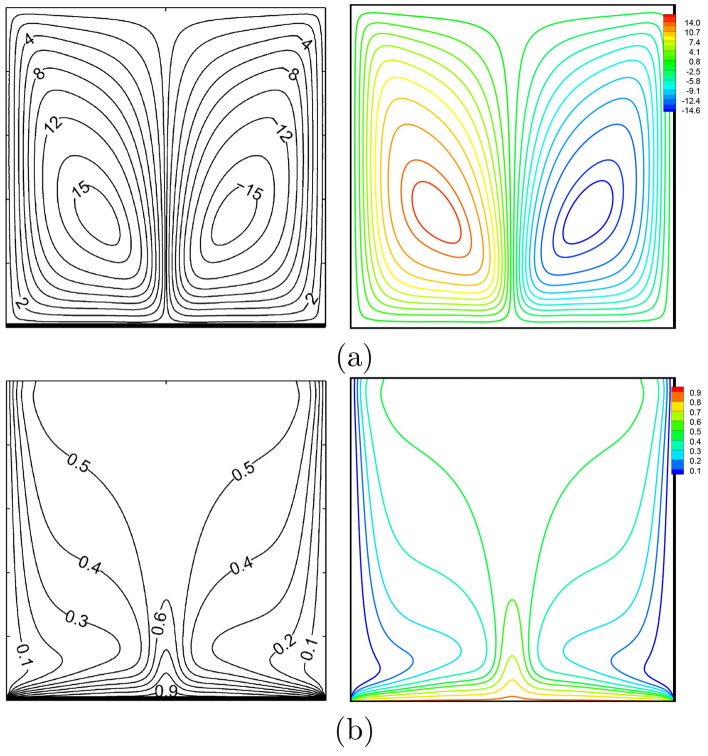
Kaluri and Basak^47^ (left) vs. present study (right): **(a)** streamlines, **(b)** isotherms ($Ra=10^{6}$, $Da=10^{-3}$, $\Pr =1000$, $N=0$, $D=1$).

**Figure 5:**
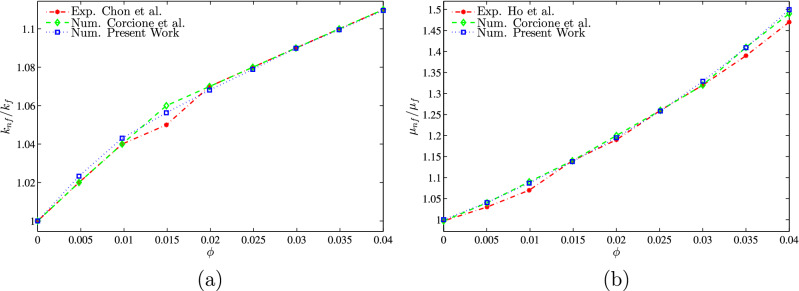
**(a)** Thermal conductivity ratio: present study vs. Chon et al.^48^ and Corcione*et al.*^49^, **(b)** dynamic viscosity ratio: present study vs. Ho et al.^50^ and Corcione et al.^49^ ($Ra=3.37\times 10^{5}$, $D=1$, $N=0$).

To validate the numerical data of the current work, the present figures were compared with the previous published numerical and experimental results presented by Calcagni et al.^46^ for the natural convection heat transfer in a square cavity heated from below, as shown in Fig. 2. In addition, Fig. 3 presents a comparison of the average Nusselt numbers between the existing work and the previous published results described by Calcagni et al.^46^. Moreover, a comparison is provided between the figures of the current work and the one indicated in Kaluri and Basak^47^ for the problem of natural convection in a square porous cavity heated from below, as described in Fig. 4. In Fig. 5, the predictions of the thermal conductivity enhancement are in agreement with the results of Chon et al.^48^, Corcione et al.^49^ and Ho et al.^50^. Except at some nanoparticle volume fractions, . The study outcomes appear to establish the validity and accuracy of the numerical approach.

## Results and discussion

This section presents numerical results for the streamlines, isotherms of the nanofluid phase, and isotherms of the solid phase for the following six parameters. These parameters are the Darcy number ($10^{-6} \le Da \le 10^{-2}$), nanoparticle volume fraction ($0\le \phi \le 0.04$), number of undulations ($0 \le N \le 4$), modified conductivity ratio ($10^{-1}\le \gamma \le 10^4$), dimensionless position of heat source ($0.2 \le B \le 0.8$) and the dimensionless length of heat source ($0.2 \le D \le 0.8$). The values of the amplitude, inter-phase heat transfer coefficient, porosity of the medium and the Prandtl number are fixed at $A=0.1$, $H=10$, $\varepsilon =0.5$ and $\Pr =4.623$, respectively. The values of the local and average Nusselt numbers are also calculated for various values of *Da*, $\phi$ and *D*. The thermos-physical properties of the base fluid (water) and solid $\mathrm {Al}_{2}\mathrm {O}_{3}$ phases are tabulated in Table 2.

**Table 2:** $\mathrm {Al}_{2}\mathrm {O}_{3}$-water’s properties at $T=310$ K^51^.

Physical properties	Fluid phase (water)	$\hbox {Al}_{2} \hbox {O}_{3}$
$C_p\, \mathrm {(J/kg K)}$	4178	765
$\rho \, \mathrm {(kg/m^3)}$	993	3970
$k\, \mathrm {(W m^{-1} K^{-1})}$	0.628	40
$\beta \times 10^{5}\, \mathrm {(1/K)}$	36.2	0.85
$\mu \times 10^{6}\, \mathrm {(kg/ms)}$	695	–
$d_p\, \text {(nm)}$	0.385	33

### Effect of Darcy number

**Figure 6:**
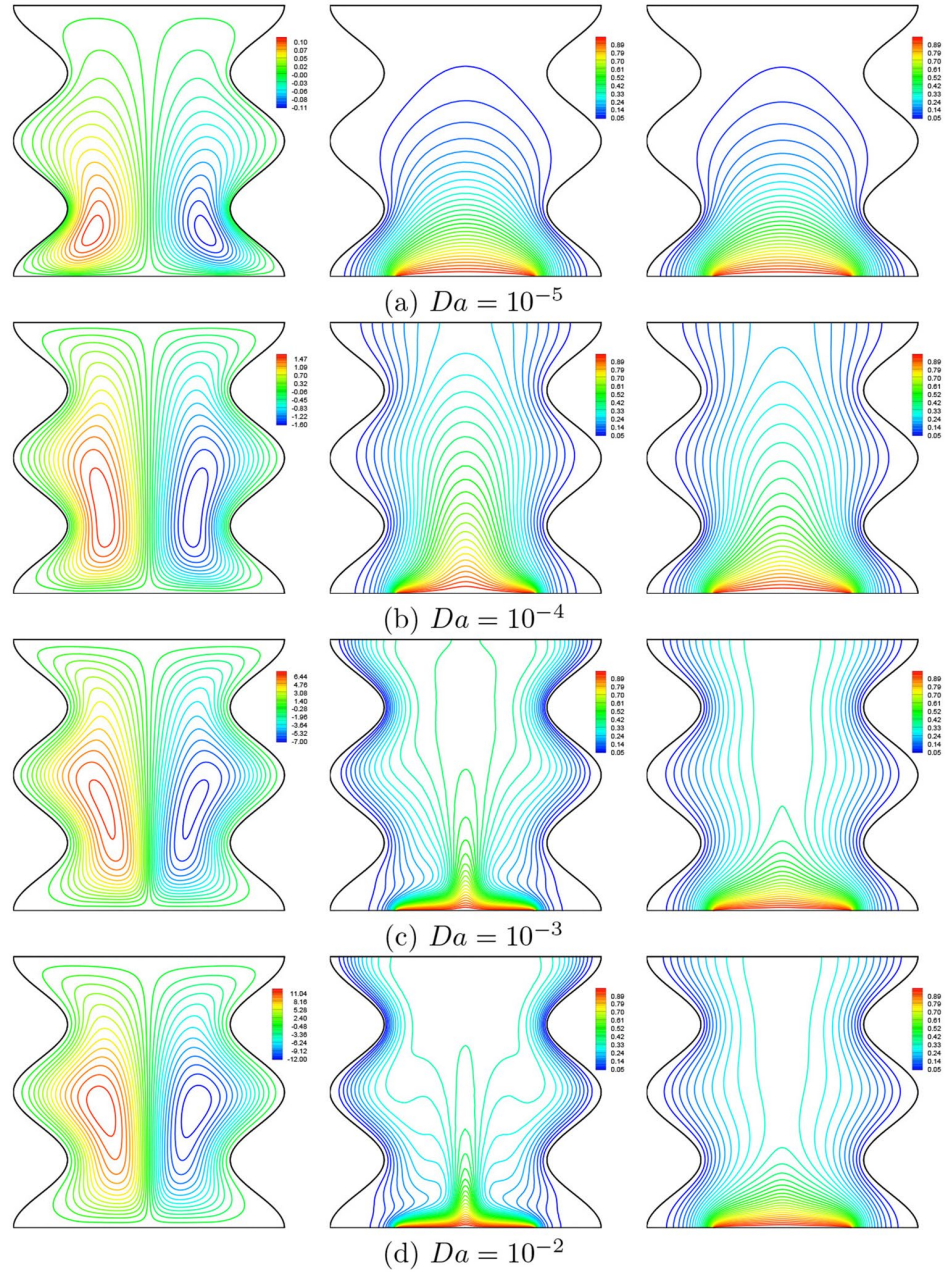
Effects of *Da* on streamlines (left), isotherms (nanofluid phase) (middle), and isotherms (solid phase) (right) ($\phi =0.02$, $N=2$, $B=0.5$, $\gamma =10$, $D=0.5$).

Figure 6 illustrates streamlines (left), isotherms of the nanofluid phase (middle) and isotherms of the solid phase (right) for $\phi =0.02$, $N=2$, $\gamma =10$, $B=0.5$ and $D=0.5$ to show the influence of Darcy number on flow and thermal fields inside the porous cavity. For a small Darcy number, the isotherms are almost parallel and therefore the heat transfer is mainly by conduction because the porous medium becomes less permeable so the speed of the flow becomes low. As a result, the porous matrix causes the flow to cease in the porous region. For the streamlines, we noticed the formation of two counter-rotating cells, one of which rotates clockwise while the second rotates anti-clockwise. The fluid that is heated by the bottom heater moves to the upper part of the cavity where it divides into two streams, one moves towards the left vertical cold wavy wall and the other moves towards the right one. When Darcy exceeds the value of $10^{-4}$, convective heat transfer begins to dominate, the isotherms are destroyed and a natural convection plume appears and the two cells take an elliptical form. The flow velocity increases because the permeability increases with increasing Darcy number, so fluid displacement in the pores is freer and the convection mode is dominant. It can be seen from this figure also that the isotherms contours within the solid phase are less destroyed due to the relatively low modified conductivity ratio ($\gamma$). It should be noted that the isotherms of the nanofluid phase and those of the solid phase are almost the same for the low Darcy number. This allows us to conclude that the local thermal equilibrium (LTE) state in the porous cavity is verified at this situation when the heat transfer mode is dominated by conduction.

**Figure 7:**
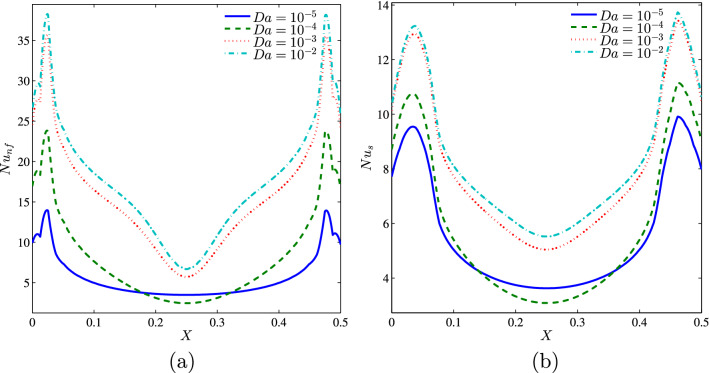
*Nu* vs. *X*: **(a)** nanofluid phase, **(b)** solid phase for several *Da* ($\phi =0.02$, $N=2$, $D=0.5$, $\gamma =10$, $B=0.5$).

The variation of the local Nusselt number for the nanofluid and the solid phase along the heated part of the bottom horizontal wall of the cavity show that the Darcy number affects the distribution of the local Nusselt number. Minimum values of the local Nusselt number are obtained in the middle of the heated part since this is where the temperature gradient is low in view of the isotherms of Fig. 6. On the other hand, the two peaks of the distribution of Nusselt numbers on the edges of the heater part correspond to the contact of the cold fluid from the side walls with the ends of the heated source, which will give rise to the highest temperature gradients. It is seen also that the values of Nusselt for the nanofluid phase are always higher than that of the solid phase (see Fig. 7a,b).

**Figure 8:**
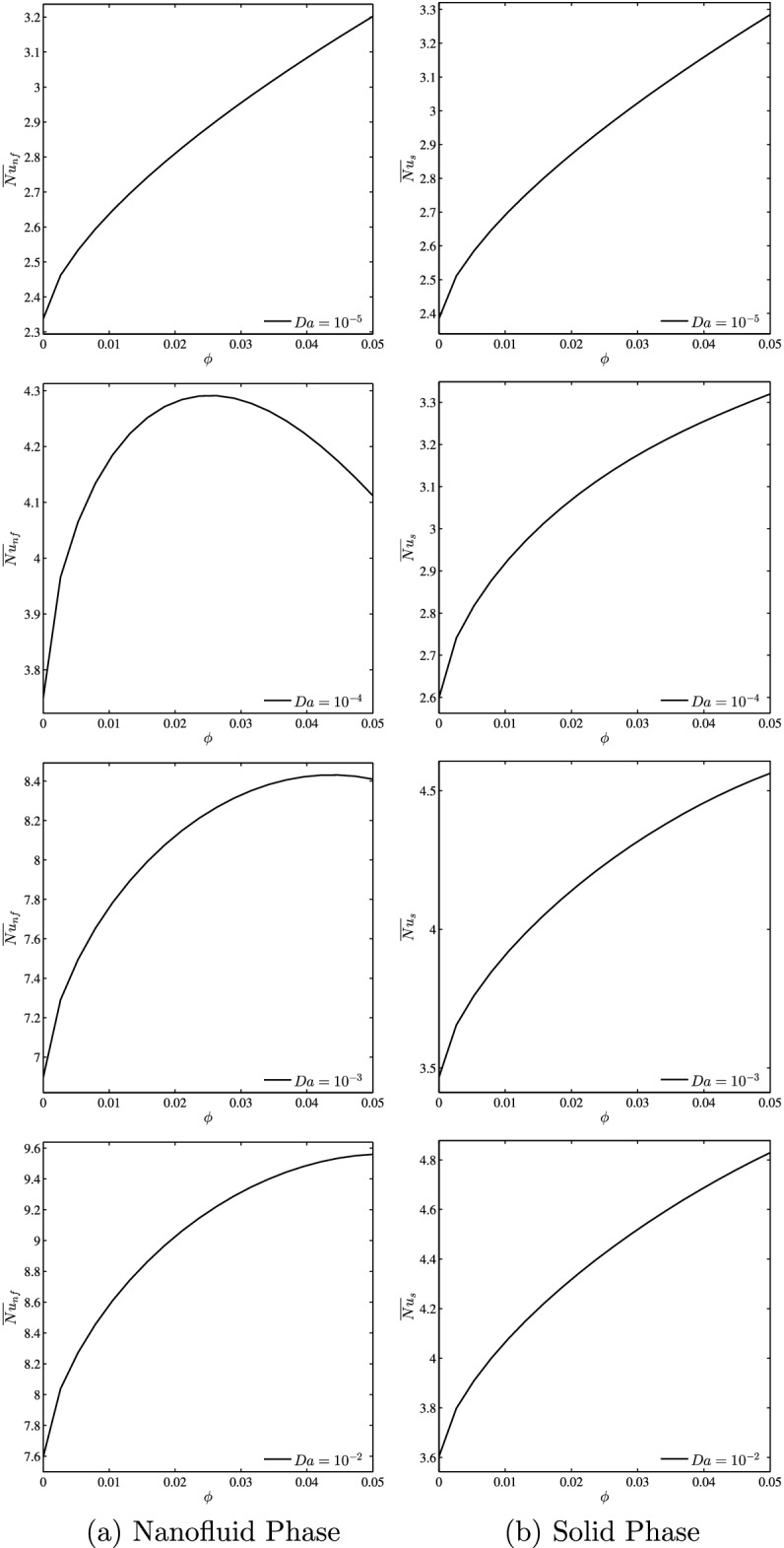
*Nu* vs. $\phi$: **(a)** nanofluid phase, **(b)** solid phase for several *Da* ($N=2$, $D=0.5$, $\gamma =10$, $B=0.5$).

Figure 8 depicts the mean Nusselt number for the nanofluid phase (a) and the solid phase (b) versus the nanoparticle volume fraction at each Darcy number separately when $N=2$, $\gamma =10$, $B=0.5$ and $D=0.5$. One observes that both Nusselt numbers continually increase with increasing values of the volume fraction of nanoparticles. This is evident because the addition of nanoparticles causes a noticeable improvement in the effective thermal conductivity of the porous media. It is remarked that when $Da \ge 10^{-4}$ there is an optimum value of nanoparticles concentration for a maximum average Nusselt number for nanofluid phase and by increasing Darcy number the maximum values of nanofluid phase average Nusselt are shifted to the high values of volume fraction of nanoparticles. Furthermore, as *Da* decreases, the fluid-to-solid Nusselt number difference decreases, indicating that the LTE state is validated in the enclosure as we reported earlier.

### Effect of nanofluid loading

**Figure 9:**
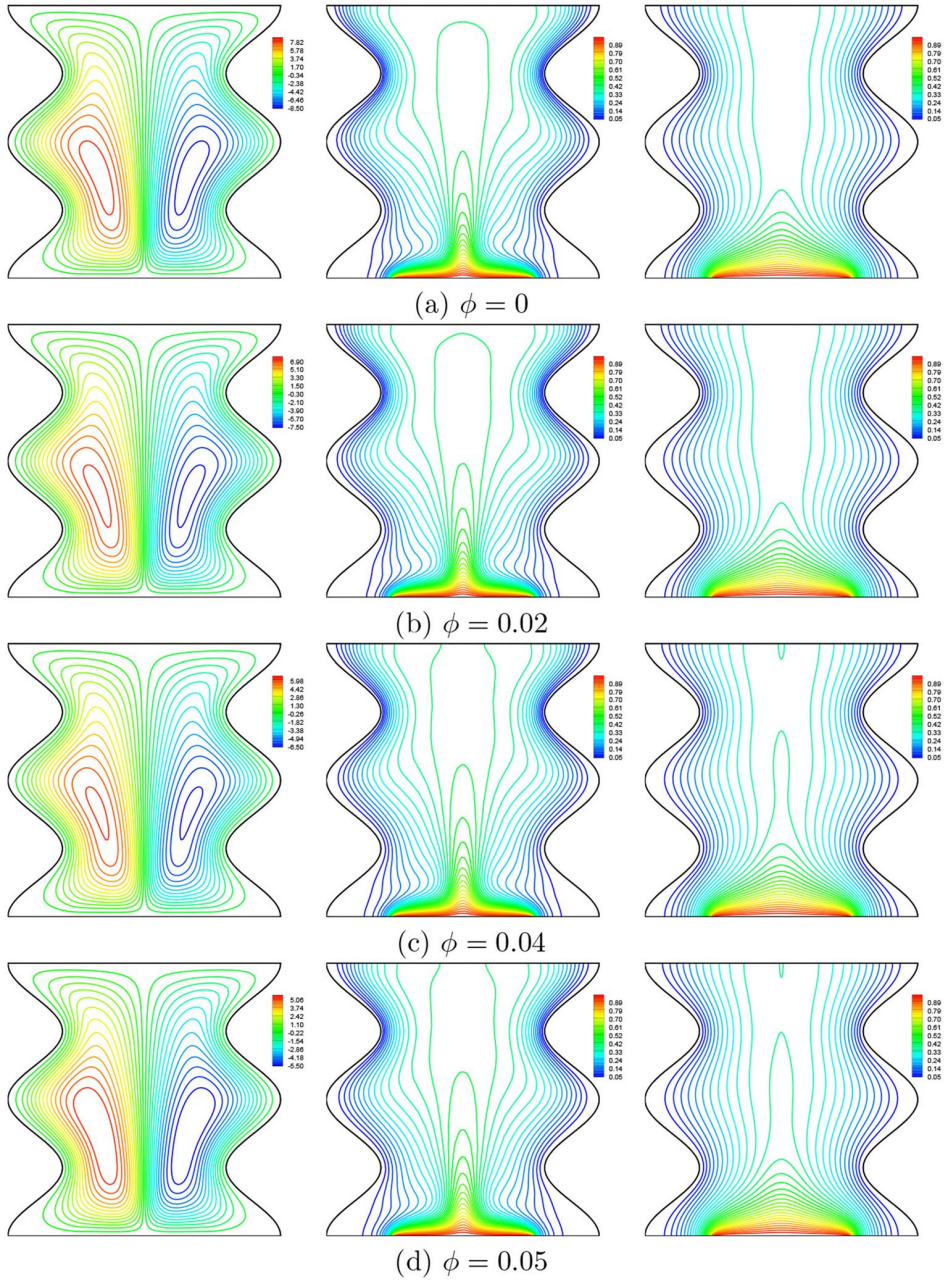
Effects of $\phi$ on streamlines (left), isotherms (nanofluid phase) (middle), and isotherms (solid phase) (right) ($Da=10^{-3}$, $N=2$, $B=0.5$, $\gamma =10$, $D=0.5$).

Figure 9 shows the effect of addition of nanoparticles on the streamlines, isotherms of the nanofluid phase and isotherms of the solid phase at $Da=10^{-3}$, $N=2$, $\gamma =10$, $B=0.5$ and $D=0.5$. The first remark is that the addition of nanoparticles leads the intensity of streamlines to decreases due to the increase in the effective viscosity of the mixture. The thermal contours depict that the distribution of the isotherms within the nanofluid phase approaching to be similar to that of the solid phase by increasing the volume fraction of nanoparticles. This is well illustrated for weak Darcy numbers where the Nusselt numbers for the two phases approach each other for $Da \le 10^{-4}$ in the stage where heat transfer by conduction dominates (See Fig. 11). In addition, it can be seen from this figure that with increase of the volume fraction from 0.01 to 0.05, the Nusselt numbers of the nanofluid phase and the solid phase increase. Furthermore, the addition of the nanoparticles greatly affects the solid phase Nusselt numbers in comparison to those of the nanofluid phase. This is because nanoparticles increase the thermal conductivity in particular which increases the rate of heat transfer when the conductive heat transfer is dominant.

**Figure 10:**
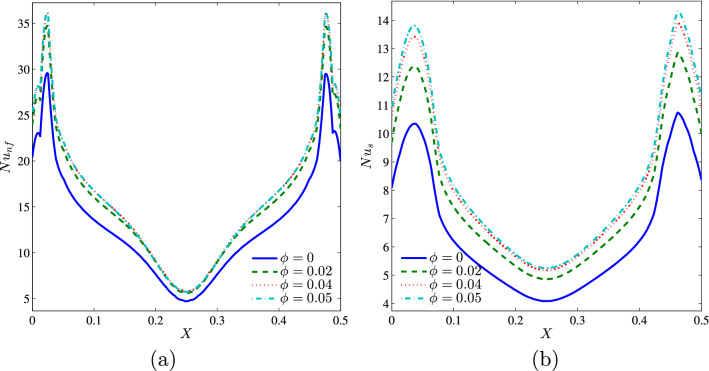
**(a)** $Nu_{nf}$ vs. *X* and **(b)** $Nu_{s}$ vs. *X* for different $\phi$ ($Da=10^{-3}$, $N=2$, $D=0.5$, $\gamma =10$, $B=0.5$).

From Fig. 10, we clearly observe that the nanoparticles concentration affects much more the distribution of the solid phase local Nusselt numbers, $Nu_s$ compared to their effect on the local Nusselt numbers for the nanofluid phase, $Nu_{nf}$ this is due to the augmentation of the thermal conductivity of the solid nanoparticles which is directly related to the solid phase thermal conductivity.

**Figure 11:**
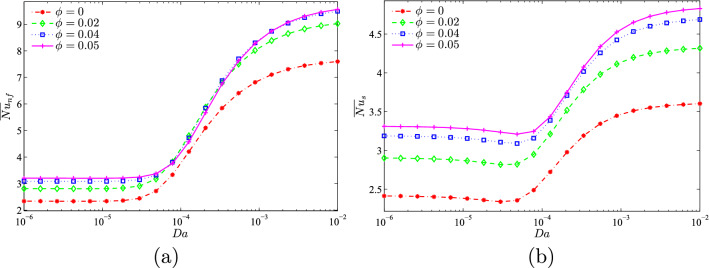
**(a)** $\overline{Nu}_{nf}$ vs. *Da* and **(b)** $\overline{Nu}_{s}$ vs. *Da* for several $\phi$ ($N=2$, $D=0.5$, $\gamma =10$, $B=0.5$).

**Figure 12:**
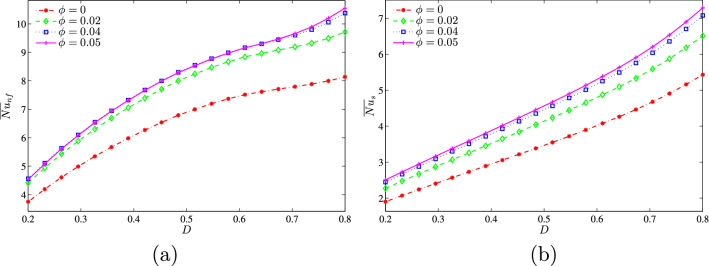
**(a)** $\overline{Nu}_{nf}$ vs. *D* and **b** $\overline{Nu}_{s}$ vs. *D* for several $\phi$ ($Da=10^{-3}$, $B=0.5$, $N=2$, $\gamma =10$).

Figure 12a, b shows that when the convective flow is strong at a relatively high *Da* value ($Da=10^{-3}$), the increase of the dimensionless length of the heat source causes an increase in the nanofluid phase and solid phase average Nusselt numbers. At this stage for a given value of *D*, the addition of nanoparticles increases the mean Nusselt number and this rate of increase is greater for low values of $\phi$.

### Effect of undulations

**Figure 13:**
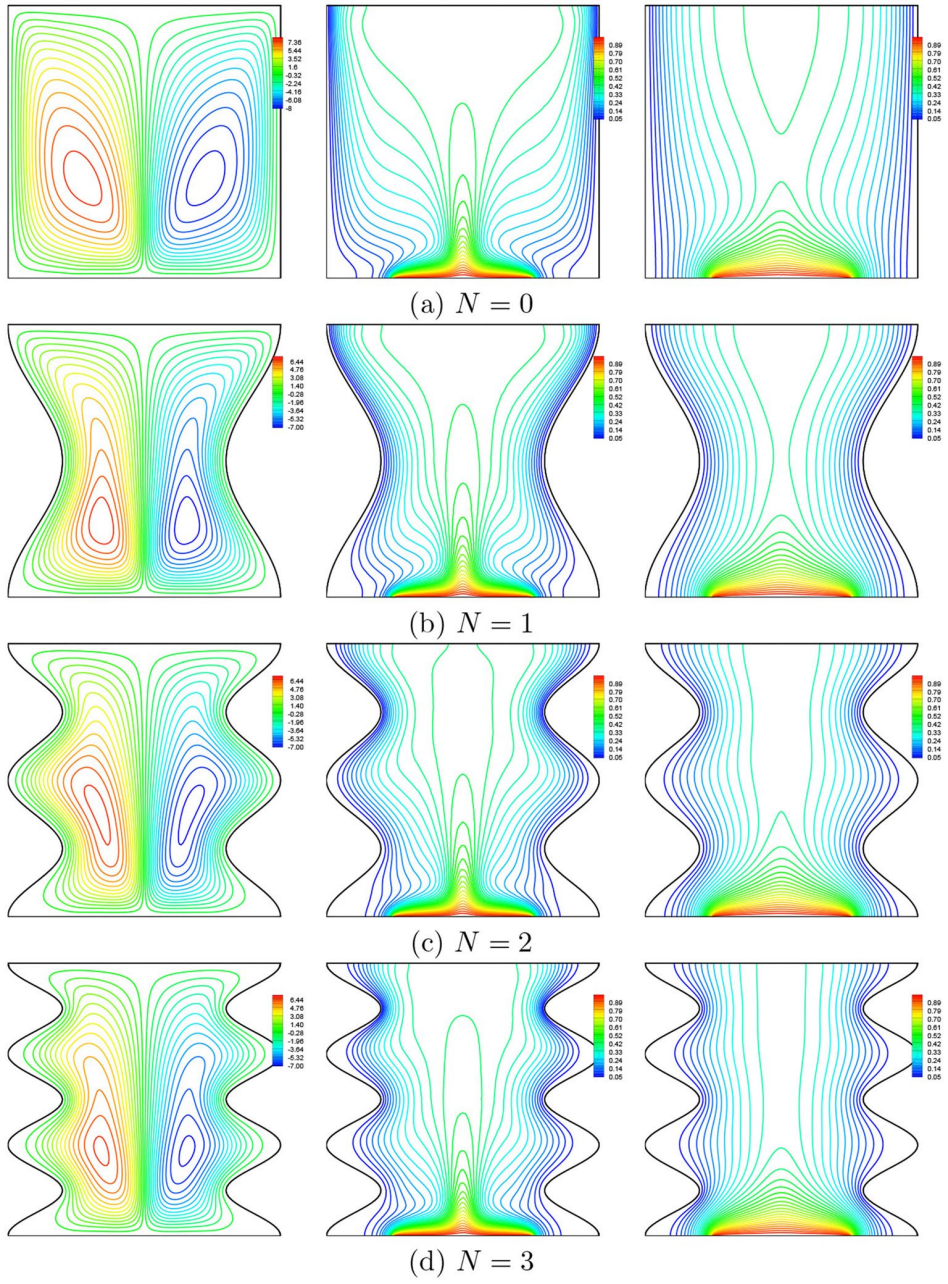
Effects of *N* on streamlines (left), isotherms (nanofluid phase) (middle), and isotherms (solid phase) (right) ($Da=10^{-3}$, $\phi =0.02$, $\gamma =10$, $B=0.5$ and $D=0.5$).

In order to investigate the effect of the of oscillations *N* (from 0 to 3) on the thermal and dynamic characteristics within the enclosure, we illustrate in Fig. 13 the streamlines, isotherms of the nanofluid phase and the isotherms of the solid phase at $Da=10^{-3}$, $\phi =0.02$, $\gamma =10$, $B=0.5$ and $D=0.5$. It appears from this figure that the presence of undulations on the vertical walls influences the geometric shape of the flow cells, as well as the distribution of isotherms near the corrugated walls. The streamlines are serried at the crests of the wave leading to an acceleration of the fluid in these areas. In addition, by increasing the oscillation number, the temperature gradient increases near the cold vertical walls especially in the upper part of these walls, so a significant heat transfer rate occurs close to these areas.

**Figure 14:**
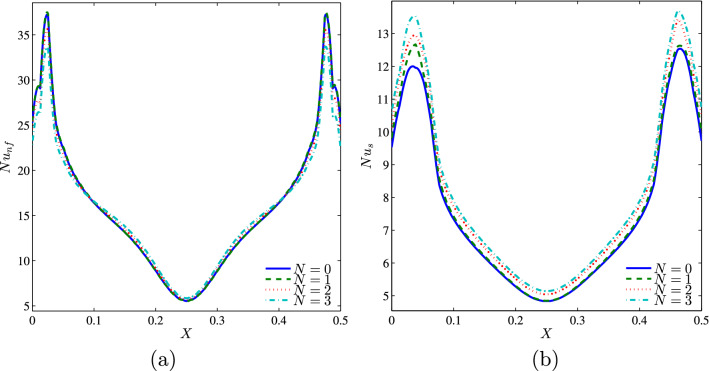
**(a)** $Nu_{nf}$ vs. *X* and **(b)** $Nu_{s}$ vs. *X* for different *N* ($Da=10^{-3}$, $\phi =0.02$, $D=0.5$, $\gamma =10$, $B=0.5$).

**Figure 15:**
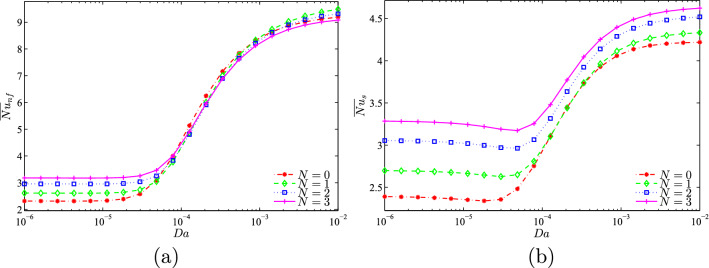
**(a)** $\overline{Nu}_{nf}$ vs. *Da* and **(b)** $\overline{Nu}_{s}$ vs. *Da* for several *N* ($\phi =0.02$, $D=0.5$, $\gamma =10, B=0.5$).

Because the corrugations on the vertical walls contribute to the reduction of natural convective flow by blocking the circulation of fluid inside the cavity, and therefore promote the thermal transfer by conduction, the solid phase Nusselt numbers at the heated part of the bottom horizontal wall increase by increasing the undulation number for all values of *Da* with a significant effect at low Darcy number. While, the increase in the number of waves, *N* enhances the nanofluid Nusselt numbers only for low Darcy numbers when the conduction is the dominant heat transfer (See Figs. 14 and 15).

**Figure 16:**
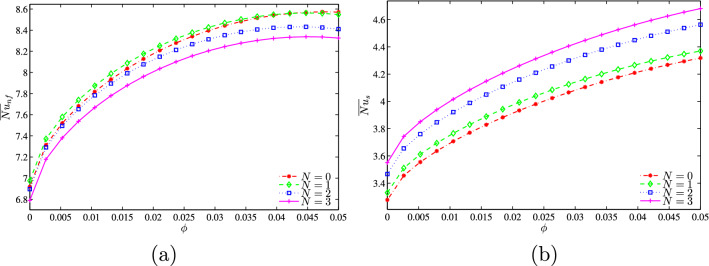
**(a)** $\overline{Nu}_{nf}$ vs. $\phi$ and **(b)** $\overline{Nu}_{s}$ vs. $\phi$ for several *N* ($Da=10^{-3}$, $D=0.5$, $\gamma =10$, $B=0.5$).

As is expected, for a relatively high Darcy number, the mean Nusselt number for the nanofluid is decreased with the increase of *N* while the corresponding one for the solid is increased as can be observed from Fig. 16.

### Effect of modified conductivity ratio

**Figure 17:**
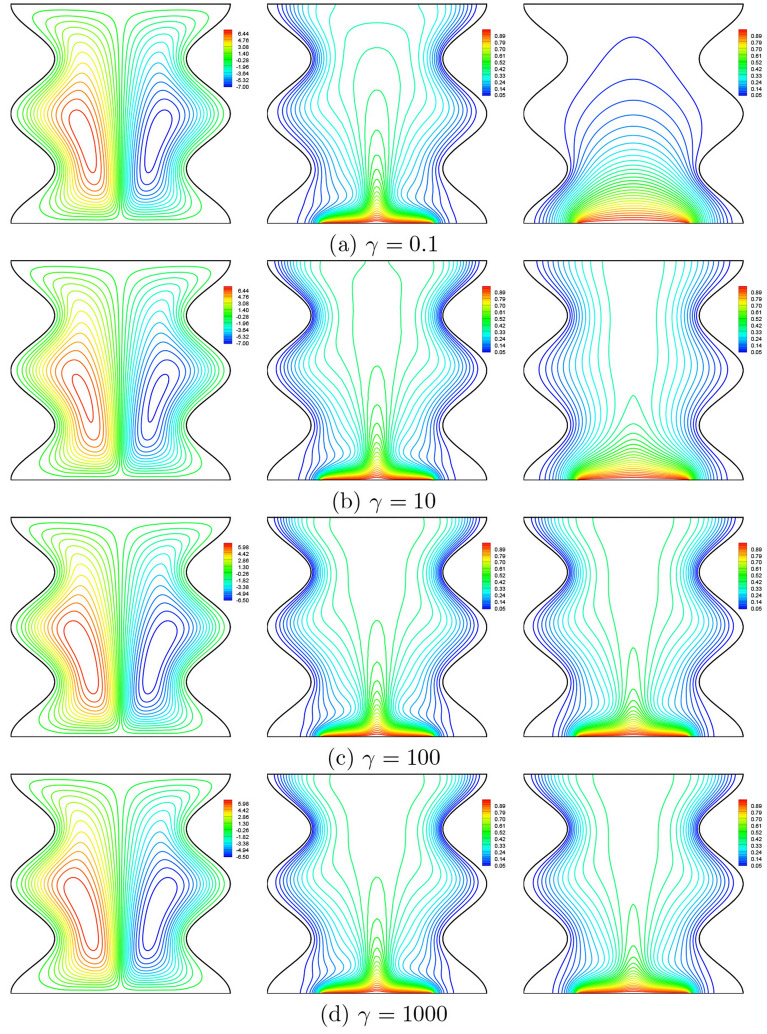
Effects of $\gamma$ on streamlines (left), isotherms (nanofluid phase) (middle), and isotherms (solid phase) (right) ($Da=10^{-3}$, $\phi =0.02$, $D=0.5$, $N=2$, $B=0.5$).

In Fig. 17, we show the effect of the modified conductivity ratio ($\gamma$) on the streamlines, isotherms of the nanofluid phase and the isotherms of the solid phase for $Da=10^{-3}$, $\phi =0.02$, $N=2$, $B=0.5$ and $D=0.5$. At this Darcy number, the modified conductivity ratio does not significantly affect the streamlines and the isotherms for the nanofluid phase, whilst the isotherms of the solid phase are considerably affected. Thus, it is seen from the isothermal contours that the local thermal equilibrium state is verified for large values of the modified conductivity ratio, which is indicated by the temperature fields for the two phases that are almost identical.

**Figure 18:**
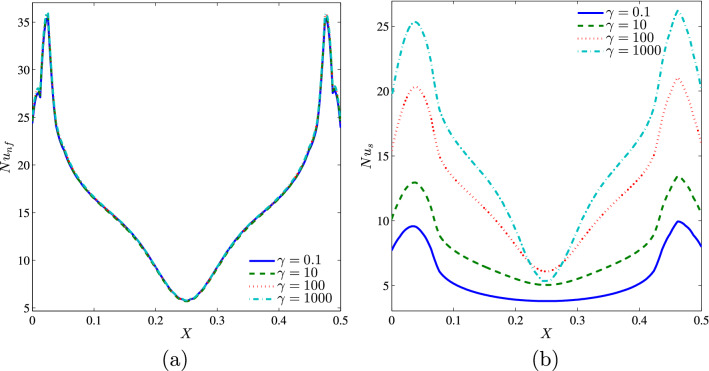
**(a)** $Nu_{nf}$ vs. *X* and **(b)** $Nu_{s}$ vs. *X* for different $\gamma$ ($Da=10^{-3}$, $\phi =0.02$, $D=0.5$, $N=2$, $B=0.5$).

**Figure 19:**
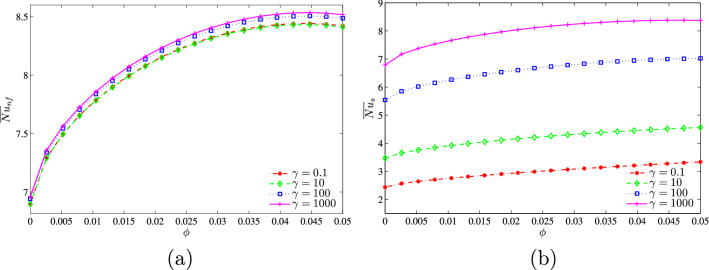
**(a)** $\overline{Nu}_{nf}$ vs. $\phi$ and **(b)** $\overline{Nu}_{s}$ vs. $\phi$ for several $\gamma$ ($Da=10^{-3}$, $B=0.5$, $N=2$, $D=0.5$).

**Figure 20:**
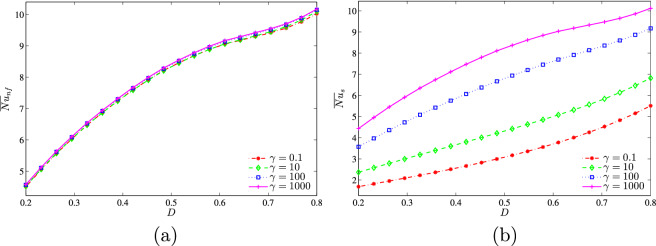
**(a)** $\overline{Nu}_{nf}$ vs. *D* and **(b)** $\overline{Nu}_{s}$ vs. *D* for several $\gamma$ ($Da=10^{-3}$, $\phi =0.02$, $B=0.5$, $N=2$).

It can be seen from Figs. 18, 19 and 20 that when the modified conductivity ratio is weak, there is a very remarkable difference between the heat transfer rates of the nanofluid and solid phases, indicating that non-equilibrium states are considerable when the modified conductivity ratio is weak. Thus, high values of the modified conductivity ratio reduce the non-equilibrium state between the porous matrix and the saturating nanofluid.

In addition, Figs. 19 and 20 show that at a given value of the modified conductivity ratio, the Nusselt numbers increase by increasing both $\phi$ and *D* and this increase is very sensitive for the solid phase, as was reported earlier.

### Effect of heater length and position

**Figure 21:**
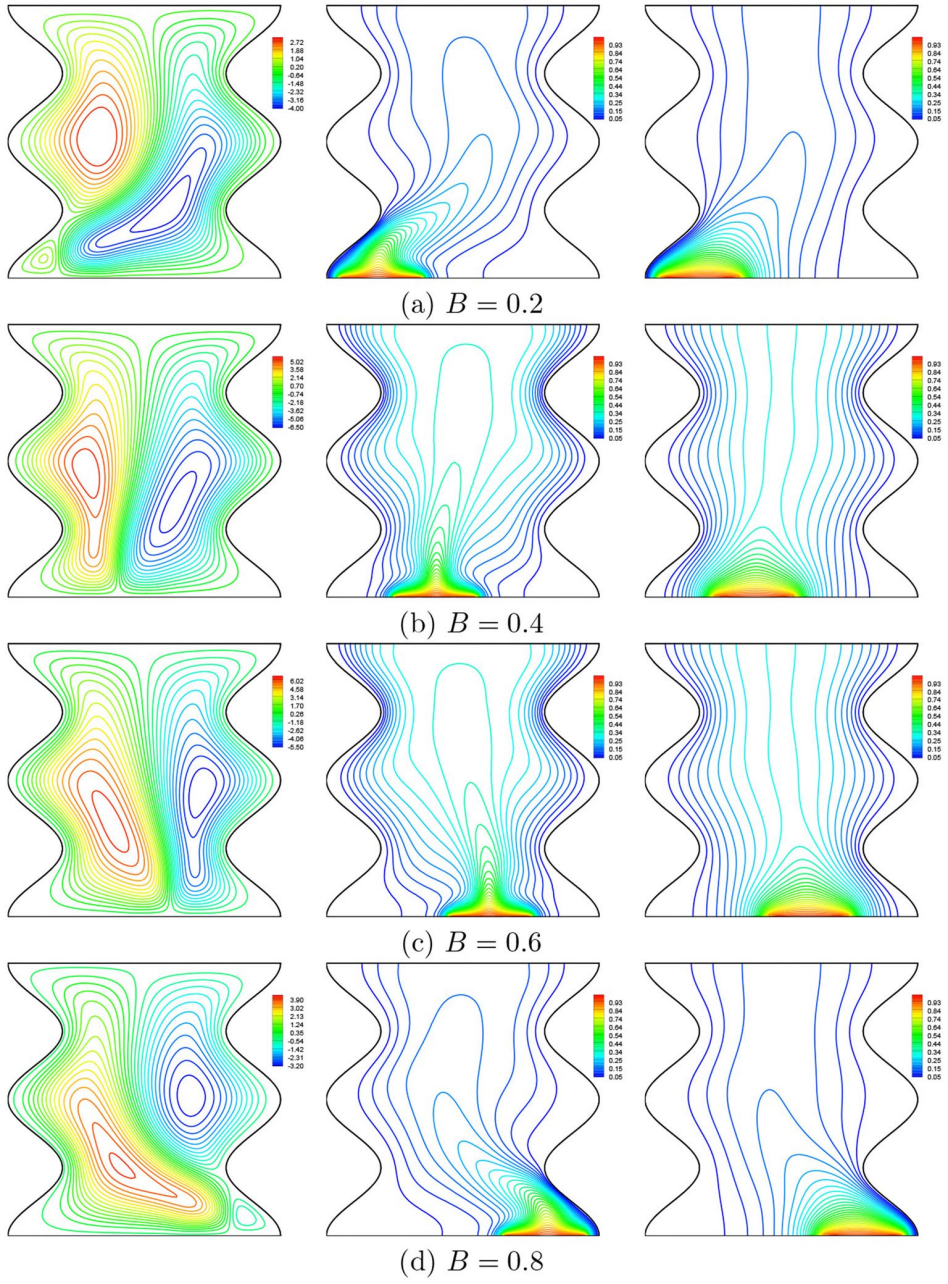
Effects of *B* on streamlines (left), isotherms (nanofluid phase) (middle), and isotherms (solid phase) (right) ($Da=10^{-3}$, $\phi =0.02$, $N=2$, $\gamma =10$, $D=0.5$).

Figure 21 shows the variations of the streamlines, nanofluid isotherms and the solid isotherms with different heat source position, *B* at $Da=10^{-3}$, $\phi =0.02$, $N=2$, $\gamma =10$ and $D=0.5$. A careful examination of this figure reveals that the displacement of the heat source to the left or to the right on the bottom wall has a major influence on the isotherms and the streamlines patterns inside the cavity. Two unsymmetrical flow vertices (with unequal strengths) and temperature fields are obtained. Moving the heater to the right increases the flow intensity in the left side of the cavity and vice versa when the heater is moved to the left that increasing the convective flow on the right side. As the heat source moves away from the side cold walls, the strength of the two flow cells increases until it reaches the middle of the bottom wall, where the two flow cells are symmetrical in the cavity.

**Figure 22:**
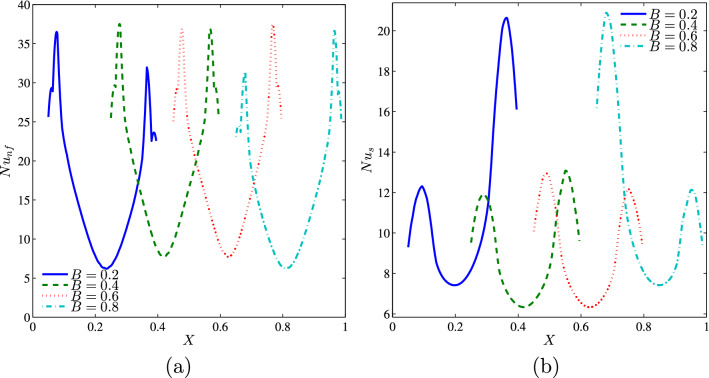
**(a)** $Nu_{nf}$ vs. *X* and **(b)** $Nu_{s}$ vs. *X* for different *B* ($Da=10^{-3}$, $\phi =0.02$, $D=0.5$, $N=2$, $\gamma =10$).

As seen from the Fig. 22, the variation of the local Nusselt number along of the heat source shows that the position of the heater affects the distribution of the solid local Nusselt numbers on the heater surface by producing a corresponding number of peaks and valleys that correspond to the maximum or the minimum heat transfer rate. Indeed, the maximum values of the solid Nusselt numbers move to the right edge of the heater when displacing it to the left and to the left edge of it when displacing it to the right (where the temperature gradient is important). On the contrary, the position of the heater does not affected much the distribution of nanofluid Nusselt numbers.

**Figure 23:**
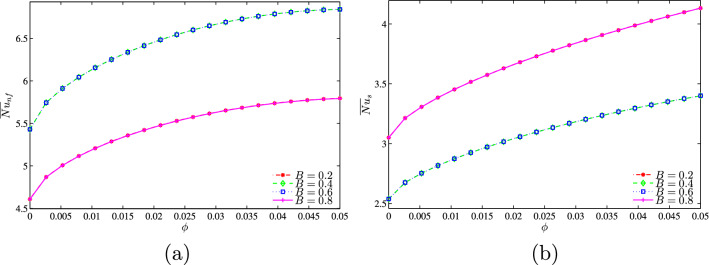
**(a)** $\overline{Nu}_{nf}$ vs. $\phi$ and **(b)** $\overline{Nu}_{s}$ vs. $\phi$ for several *B* ($Da=10^{-3}$, $D=0.5$, $N=2$, $\gamma =10$).

As expected, when displacing the heat source, the convection flow is reduced due to the unsymmetrical flow. Thus, we note a decrease in the average Nusselt numbers of the nanofluid phase while the average Nusselt numbers of the solid phase increase. It is to be noted that moving the hot source (to the left or to the right with the same degree) only affects the distribution of the local Nusselt numbers while the mean Nusselt numbers are not affected (Fig. 23). This is due to the fact that the hot source is very close in this case which makes the heat transfer so fast.

**Figure 24:**
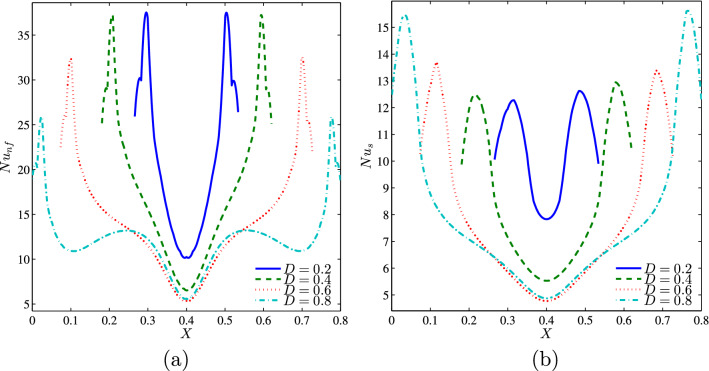
**(a)** $Nu_{nf}$ vs. *X* and **(b)** $Nu_{s}$ vs. *X* for different *D* ($Da=10^{-3}$, $\phi =0.02$, $B=0.5$, $N=2$, $\gamma =10$).

Figure 24 illustrates symmetrical profiles of the nanofluid and the solid local Nusselt numbers along the heater with different lengths. It can be seen that in the convection dominated situation, increasing the heater size causes the local Nusselt numbers profile to decrease. This is because as the heater size increases, the temperature of the heater increases due to the higher heat flux generated and consequently, the corresponding Nusselt number decreases.

**Figure 25:**
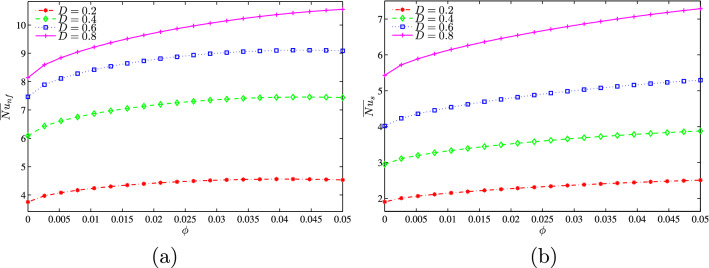
**(a)** $\overline{Nu}_{nf}$ vs. $\phi$ and **(b)** $\overline{Nu}_{s}$ vs. $\phi$ for several *D* ($Da=10^{-3}$, $B=0.5$, $N=2$, $\gamma =10$).

Evolution of the average solid and nanofluid Nusselt numbers as a function of the volume fraction of the nanoparticles for different values of D is represented on the graphs of Fig. 25a,b at $Da=10^{-3}$, $N=2$, $\gamma =10$ and $B=0.5$. In general, for given values of *D*, the average Nusselt number, whether of the solid phase or of the nanofluid phase, increases as $\phi$ increases. We also note that the two mean Nusselt numbers increase with the increase in the heater length. This can be explained by the higher heat generation rates as the heater size increases which clearly increases the area exposed to the heat transfer.

## Conclusions

Several significant conclusions drawn from this study are listed in the following:

1. When the Darcy’s numbers are small, the conduction heat transfer takes place. Once the Darcy number exceeds the value of $10^{-4}$, the convective heat transfer begins to dominate.
2. The local thermal equilibrium (LTE) state in the porous cavity is verified at low Darcy numbers when the heat transfer mechanism is dominated by conduction.
3. High values of the modified conductivity ratio, $\gamma$ reduces the non-equilibrium state between the porous matrix and the saturating nanofluid.
4. Displacing the heater from the side walls towards the middle of the bottom wall, enhances the strength of natural convection flow within the enclosure. Also, when the heater remains in the middle of the bottom wall, symmetrical flow cells are generated regardless of the heater length.
5. The presence of waves on the vertical walls leads to the reduction of the overall heat transfer when the convection is the dominant heat transfer mechanism whereas it enhances when the heat transfer mode is dominated by conduction.
6. It is found that the heater length, its position, and the number of waves on the side vertical walls as well as the nanoparticles concentration can be control parameters for heat transfer and fluid flow within the cavity.
